# Improvement of Anther Culture to integrate Doubled Haploid Technology in Temperate Rice (*Oryza sativa* L.) Breeding

**DOI:** 10.3390/plants11243446

**Published:** 2022-12-09

**Authors:** Csaba Lantos, Mihály Jancsó, Árpád Székely, Éva Nagy, Tímea Szalóki, János Pauk

**Affiliations:** 1 Department of Biotechnology, Cereal Research Non-Profit Ltd., P.O. Box 391, H-6701 Szeged, Hungary; 2 Research Center for Irrigation and Water Management, Institute of Environmental Sciences, Hungarian University of Agriculture and Life Sciences, Anna-liget 35, H-5540 Szarvas, Hungary; 3 Oud’s Amazone Trading Pty Ltd., Risleys Hill Road, Federal, NSW 2480, Australia

**Keywords:** androgenesis, anther culture, haploid, *Oryza sativa* L., field evaluation

## Abstract

Doubled haploid (DH) plant production, such as anther culture (AC), is an effective tool used in modern rice breeding programs. The improved efficient protocols applied can shorten the process of breeding. The effect of combinations of plant growth regulators (2.5 mg/L NAA, 1 mg/L 2,4-D and 0.5 mg/L kinetin; 2 mg/L 2,4-D and 0.5 mg/L BAP) in the induction medium were compared in AC for five rice breeding materials and combinations. Induction of calli ranged from 264.6 ± 67.07 to 468.8 ± 123.2 calli/100 anthers in AC of rice genotypes. Two basal media (MS and N_6_) and two combinations of growth regulators (1 mg/L NAA, 1 mg/L BAP and 1 mg/L kinetin; 1.5 mg/L BAP, 0.5 mg/L NAA and 0.5 mg/L kinetin) were used as regeneration media. The in vitro green plant production was the highest with the application of the N_6_NDK induction medium (NAA, 2,4-D and kinetin) and the MS-based regeneration medium (1 mg/L NAA, 1 mg/BAP and 1 mg/L kinetin) in anther culture of the ‘1009’ genotype (95.2 green plantlets/100 anthers). The mean of five genotypes was 24.48 green plantlets/100 anthers for the best treatment. Flow cytometric analyses conducted identified the microspore origin of the haploid calli produced in AC, while the uniformity of spontaneous DH plants was checked in the DH_1_ and DH_2_ generations. Spontaneous chromosome doubling ranged from 38.1% to 57.9% (mean 42.1%), depending on the breeding source. The generated and selected DH lines were tested in micro- and small-plot field experiments to identify promising lines for a pedigree breeding program. The improved AC method was integrated in a Hungarian temperate rice pedigree breeding program.

## 1. Introduction

Biotechnological methods support the quick and efficient production of new varieties and hybrids in crop plants. One of these techniques is anther culture (AC) which can reduce the breeding time of new varieties and hybrids due to the production of homozygous lines within one generation. In rice, AC was applied to develop new varieties in many breeding programs [1,2,3,4,5]. Research groups have focused on the further improvement of AC in rice [6,7,8,9,10,11,12,13,14,15].

Since the development of AC in rice [16], many efforts have been made in several countries to improve the efficiency of AC [17,18,19,20]. However, several factors limit the high frequency of doubled haploid (DH) plant production in rice, such as the genotype and the growing condition of donor plants, the developmental stage of microspores, pre-treatments, and the induction and regeneration media used.

Many studies have demonstrated the effect of genotype on the efficiency of AC in rice [2,21,22,23,24,25]. The japonica genotypes are known as high-responding rice genotypes, while indica genotypes are known to be less responsive to AC [6,13,21,22,24,26,27,28]. A low frequency of plant regeneration and albinism are also known as limiting factors for AC in rice [6,8,14,15,19,21,22,25].

Several studies have focused on the improvement of culture conditions in rice AC. The same medium was applied for induction of androgenesis and plant regeneration in a one-step method [3,29], while other protocols used different media for induction and plant regeneration in rice AC. The N_6_ medium [30] has been the most frequently used medium for the induction of androgenesis over the past two decades [5,6,7,8,10,11,12,13,14,21,22,23,24,25,27,31,32]. Maltose is considered the best carbon source for the induction of androgenesis and has a positive effect, increasing callus induction and green plantlet regeneration in AC [12,20,21]. Different growth regulators, such as 1-naphtylacetic acid (NAA), phenylacetic acid, Picloram, Dicamba, 2,4-dichlorophenoxyacetic acid (2,4-D) and their combinations, were applied for the induction of androgenesis in rice AC [23]. The combination of 2,4-D and kinetin has been frequently applied in the induction media of rice AC [5,7,14,22,24], while other investigations have used a combination of indol-3-acetic acid (IAA) and kinetin [10]. In indica rice, a combination of 2,4-D and 6-benzylaminopurin (BAP) was used to increase the efficiency of androgenesis induction in AC [11,12], while Mayakaduwa and Silva [13] applied a combination of 2,4-D, NAA and kinetin. A combination of NAA, BAP and kinetin was also applied for induction of androgenesis in japonica and japonica × indica crossing combinations [28,33].

A large number of calli can be induced in AC for some rice genotypes, improving plant regeneration efficiency for DH plant production. Two basal media, namely MS [11,12,13] and N_6_ [5,7,10,14], were applied mainly to achieve plant regeneration of AC-derived calli. According to published protocols, different combinations of growth regulators (BAP, IAA, NAA and kinetin) were successfully applied to achieve increased plantlet regeneration [5,7,8,10,11,12,13,14,21,22,23,24,27,31]. Some published data are available on the effects of exogenous growth regulators during plant regeneration of rice AC-derived calli [11,12]. The authors of these studies reported that the combination of 1.5 mg/L BAP, 0.5 mg/L NAA and 0.5 mg/L kinetin positively affected green plantlet production of AC-derived calli [11,12].

The aims of the present study were the improvement of in vitro AC in rice and the integration of the method into rice breeding. In the experiments undertaken, androgenesis was induced in AC of five rice genotypes to investigate the effect of genotypes on in vitro androgenesis. Different combinations of growth regulators (2 mg/L 2,4-D and 0.5 mg/L BAP–N_6_DB; 2.5 mg/L NAA, 1 mg/L 2,4-D and 0.5 mg/L kinetin–N_6_NDK) and of induction medium were tested on the AC parameters (i.e., number of AC-derived calli, regenerated green and albino plantlets). In the plant regeneration, two basal media (MS and N_6_) and two combinations of growth regulators (1 mg/L NAA, 1 mg/L BAP and 1 mg/L kinetin–NBK1; 1.5 mg/L BAP, 0.5 mg/L kinetin and 0.5 mg/L NAA–NBK2) were compared to identify the most efficient plant regeneration medium. The percentage of spontaneous chromosome doubling was determined based on seed production. In addition, DH lines in the DH_1_ and DH_2_ generations were selected in micro- and small-plot field experiments examining plant height, biomass, yield, lodging, blast resistance, TKW, and quality parameters, to identify promising lines for the Hungarian temperate rice breeding program.

## 2. Results

### 2.1. Effect of Genotype, Growth Regulators and Their Interactions on Androgenesis Induction in AC

**Figure 1:**
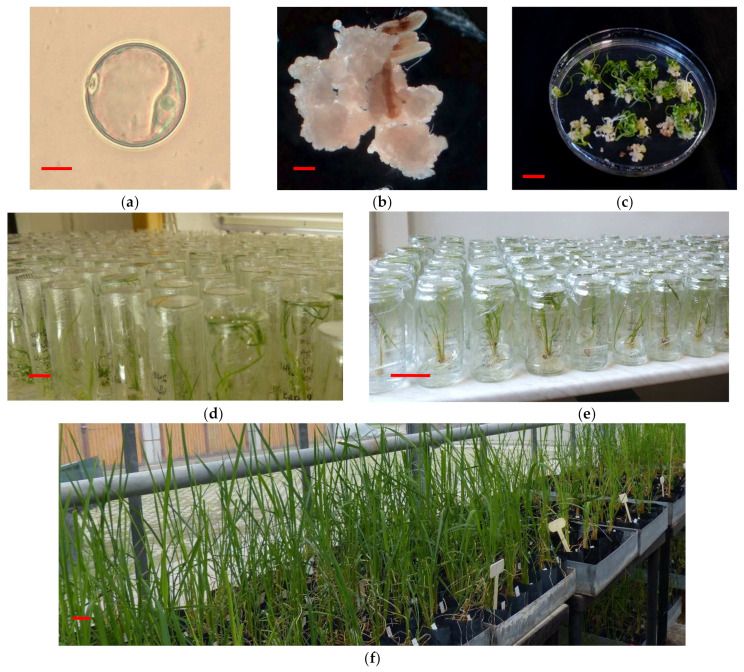
The AC of rice: (**a**) uninucleated microspore, (**b**) microspore-derived calli in AC of rice. (**c**) Plant regeneration of AC-derived calli from a high responding genotype. (**d**) Rooting of AC derived green plantlets in individual tubes. (**e**) Developing green plantlets in individual jars. (**f**) Acclimatized plants in the glasshouse. Bars = 10 µm for (**a**); 1 mm for (**b**); 10 mm for (**c**) and (**d**); 50 mm for (**e**) and (**f**).

The growth regulators significantly influenced the number of microspore-derived calli (Figure 1a,b), while the effect of genotype and the interaction of genotype and the growth regulators were not significant (Table 1). Numerous AC-derived calli were produced in each treatment; the production of calli was genotype-independent. In AC of rice genotypes using the N_6_NDK induction medium, 468.80, 376.80, 333.60, 320.00 and 264.60 calli/100 anthers were produced in AC of Karola, M × K, 1009, Á × D, and Dáma, respectively.

The effect of combinations of growth regulators (2 mg/L 2,4-D and 0.5 mg/L BAP–N_6_DB; 2.5 mg/L NAA, 1 mg/L 2,4-D and 0.5 mg/L kinetin–N_6_NDK) was compared according to the number of AC-derived calli produced for five rice genotypes (Table 2). Androgenesis induction was effective for all treatments. The number of AC-derived calli ranged from 264.6 calli/100 anthers (Dáma) to 468.8 calli/100 anthers (Karola) in the best treatment (N_6_NDK induction medium). All genotypes produced a higher quantity of calli on the N_6_NDK induction medium than on the N_6_DB medium. These differences were significant for three breeding sources (Karola, 1009 and M × K).

### 2.2. Determination of Ploidy Level of AC-Derived Rice Calli

**Figure 2:**
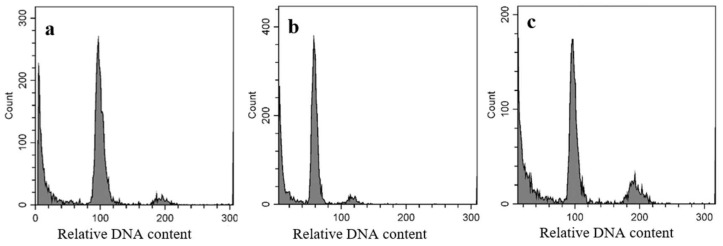
Flow cytometric analyses of rice calli: histograms demonstrate the relative DNA content of (**a**) somatic tissue culture-derived callus, diploid control; (**b**) AC-derived haploid callus; (**c**) AC-derived diploid callus.

The ploidy levels of 10 AC-derived calli were determined using flow cytometric analyses. The measurements revealed the relative DNA content (ploidy level) of the samples. Figure 2 demonstrates the ploidy level of the AC-derived calli compared to that of the callus propagated in the somatic tissue culture (control, Figure 2a). Based on the histogram of flow cytometric analyses, four haploid (Figure 2b) and six diploid calli (Figure 2c) were identified among the tested calli.

### 2.3. Regeneration of Green and Albino Plantlets from AC-Derived Calli

The AC-derived calli induced on the two induction media were used to test the plant regeneration efficiency. The AC-derived calli were transferred parallel to the four different regeneration media (N_6_NBK1, N_6_NBK2, MSNBK1, MSNBK2). The effects of the basal media and growth regulators were tested based on the number of regenerated green and albino plantlets. The genotype and regeneration medium significantly influenced the number of green and albino plantlets (Table 3). Genotype and medium interactions were significant in the production of green plantlets. Figure 1c illustrates that the tested genotypes produced green and albino plantlets.

The mean of the green plantlets regenerated was higher from the calli induced on the N_6_NDK induction medium (13.68 green plantlets/100 anthers) than on the N_6_DB induction medium (11.76 green plantlets/100 anthers). The average numbers of the regenerated albino plantlets were 18.76 and 12.32 by 100 anthers on the N_6_NDK and N_6_DB induction media, respectively (Table 4).

The positive effect of the MS basal medium on the number of regenerated green and albino plantlets was clear compared with the N_6_ medium (Table 4). The highest efficiency of green plantlet production (24.48 green plantlets/100 anthers, average of five genotypes) was achieved for the combination of the MSNBK1 plant regeneration medium with AC-derived calli induced on the N_6_NDK induction medium. The plant regeneration efficiency was highest (95.2 green plantlets/100 anthers) for the best treatment of the 1009 genotype (Table 4). The least effective green plant regeneration method (4.8 green plantlets/100 anthers, average of five genotypes) was observed using a combination of the N_6_DB induction medium and the N_6_NBK2 plant regeneration medium.

The number of albino plantlets ranged from 0 albinos/100 anthers to 52.8 albinos/100 anthers, depending on genotype and treatment. Based on the data for five genotypes, the mean albino production (28.32 albino plantlets/100 anthers) was higher than mean green plantlet production (24.48 green plantlets/100 anthers) for the most effective regeneration method (N_6_NDK induction medium and MSNBK1 plant regeneration medium). However, this did not prevent the highly successful production of hundreds of AC-derived green plantlets for further breeding and applied research purposes.

**Figure 3:**
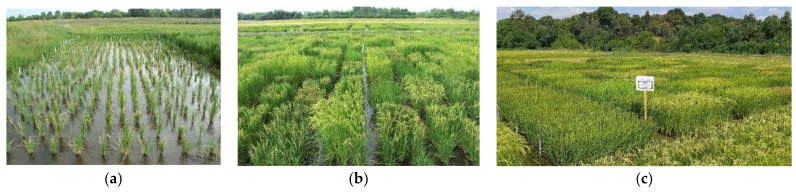
Propagation and evaluation of DH lines in the nursery: (**a**) growing of acclimatized DH_0_ plants in field conditions, (**b**) observation of phenotypic and agronomic characters of DH lines (DH_1_) in micro-plots, (**c**) yield trial experiment of DH lines (DH_2_) in the small-scale field evaluation program.

The regenerated green plantlets were grown first in individual tubes and later in jars until transplantation of well-developed green plantlets into the greenhouse (Figure 1d,e,f). After acclimatization, the plants were transplanted to the field (Figure 3a), where the spontaneous DH plants produced seeds while the haploid plants remained sterile. The highest number of DH plants (234) were identified for the ‘M × K’ genotype (Table 5). One hundred and sixty-nine plants were found as DH in the 1009 line while ‘Á × D’, Dáma and Karola produced 18, 11 and 9 DH plants, respectively. The genotype influenced the number of spontaneous DH plants. Altogether, 441 mature DH plants were identified among the regenerants; the mean spontaneous chromosome doubling was 42.1%, ranging from 38.1% to 57.9% depending on genotype. The developmental and morphological characters of single plants were determined and compared according to special selection criteria (i.e., early flowering 70–90 DAS, 75 cm maximum plant height, no lodging, awnless seeds, freedom from blast disease). The seeds of DH plants were collected and integrated into the breeding program.

**Figure 4:**
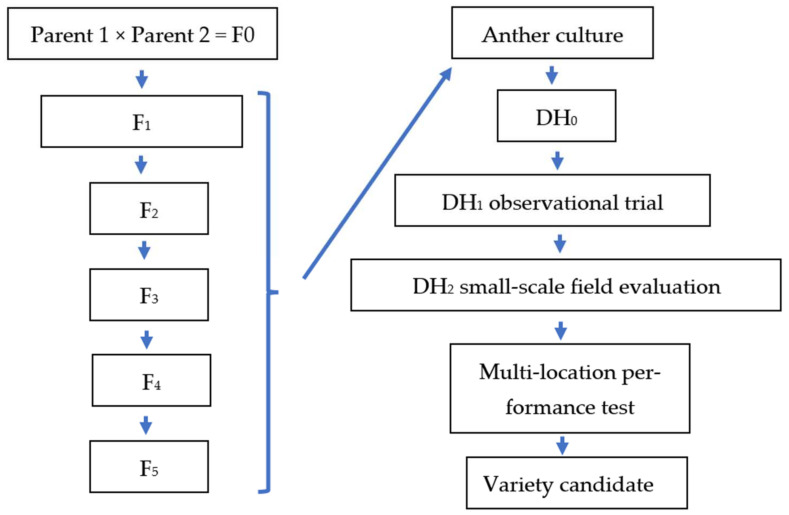
Integration of in vitro AC method into rice breeding program in Hungary.

Figure 4 shows the integration of AC method in temperate rice breeding program, which can be used for different generations of the pedigree breeding process.

### 2.4. Field Evaluation and Integration of DH-Lines in Breeding Program

In 2018, the seeds of individual DH_0_ plants were sown into micro-observation plots to evaluate basic phenotypic and agronomic characteristics (Figure 3b). The homozygosity of DH lines was also confirmed in the DH_1_ generation. The ratio of valuable DH lines was lower among the DH lines derived from the F_1_ generation in comparison with the advanced lines (Table 6). Based on the phenotypic parameters (i.e., plant height, plant habit, kernel shape, flowering time) and agronomic traits (i.e., cold sensitivity, lodging, blast resistance, shattering), 10 DH lines were selected from the observation plots. These were advanced to replicated yield trials for continued evaluation. In the DH generation, DH lines were tested in the small-scale field evaluation program (Figure 3c, Table 7 and Table 8) for two consecutive years (2019–2020). Seasonal effects were significant only in the case of milling quality (i.e., whole and broken white rice); therefore, the other traits were evaluated without splitting yearly data.

Based on the complex evaluation (Table 7 and Table 8), six DH_2_ lines were selected for further experiments or breeding components (as promising lines). ‘Tünde 16.II. 1’ was the best performing genotype with the highest yield and resistance to blast. Five genotypes had higher average yield compared to the mean yield for the control varieties. These promising lines were transferred for further field evaluation trials along with conventional breeding lines for further comparisons and integration in the temperate rice pedigree breeding process.

## 3. Discussion

DH plant production methods are excellent tools for breeding programs and applied crop plant research to enhance the improvement of new genotypes. Therefore, the improvement of DH plant production has remained the focus of rice breeding and research [6,7,8,9,10,11,12,13,14,23,24,28,32].

### 3.1. Induction of Androgenesis in Rice

Since the first reports of rice AC-derived plants [16], several bottlenecks (e.g., contamination, genotype dependency, low plant regeneration efficiency and albinism) have been highlighted [5,6,8,19,21,22,25,34]. 

A large quantity of calli on the N_6_NDK induction medium was produced, independent of genotype, in AC of the five tested japonica genotypes, while there have been some reports of an effect of genotype on the number of produced calli [14,25,33]. Hence, the improvement of culture conditions and media composition can mitigate genotype dependency in AC. Other studies have found that the efficiency of in vitro androgenesis was influenced by the exogenous growth regulators used [11,12,14,23]. In AC of an indica rice hybrid, Naik et al. [12] reported that the combination of 2 mg/L 2,4-D and 0.5 mg/L BAP in the induction medium had a positive effect on androgenesis compared with other combinations of growth regulators (i.e., 2,4-D, kinetin, BAP, NAA, TDZ). In this study, a combination of 2,4-D and BAP (N_6_DB) was compared with a combination of NAA, 2,4-D and kinetin (N_6_NDK) [32]. The combination of 2.5 mg/L NAA, 1 mg/L 2,4-D and 0.5 mg/L kinetin positively affected the callus induction rate and regeneration of green and albino plantlets.

### 3.2. Effect of the Basal Medium on Plantelt Regeneration

In the last decade, two different basal media have been frequently cited for plant regeneration, namely, MS [8,11,12,32] and N_6_ [5,7,10,14]. In an experiment undertaken by us, the two media were compared based on the plant regeneration efficiency. The superiority of the MS medium was based on the number of regenerated green and albino plantlets. Two to three times more green plantlets were regenerated with application of the MS basal medium. Furthermore, these differences were even more marked based on the number of albino plantlets regenerated.

### 3.3. Effect of Growth Regulators on the Plantlet Regeneration

A wide range of growth regulators (BAP, IAA, NAA and kinetin), and their combinations, were applied to induce green plantlet production in rice AC. However, efficient green plantlet production proved to be a critical issue, especially in AC of the indica type [6,21,22,24,26,27,28]. A low frequency of plant regeneration and albinism affected the production of the DH lines [9,11,12,14,28,32].

A combination of NAA, BAP and kinetin has been frequently applied for plant regeneration of AC-derived rice calli [11,12,21,23,24,32]. Some researchers have also investigated combinations of kinetin and NAA [5,10,14,27], kinetin, NAA and IAA [7] or BAP and NAA [22]. Few studies are available which have focused directly on a comparative analysis of growth regulators during the plant regeneration period [23].

Researchers from India tested different combinations of BAP, kinetin and NAA on plantlet regeneration efficiency in indica rice hybrids [11,12]. In these experiments, the highest plant regeneration frequency was achieved with a combination of 1.5 mg/BAP, 0.5 mg/kinetin and 0.5 mg/L NAA. In the present experiment, both tested combinations of growth regulators were effective for producing green plantlets. However, the highest green plantlet production efficiency (24.48 green plantlets/100 anthers, average of five genotypes) was achieved on the MS regeneration medium supplemented with 1 mg/L NAA, 1 mg/L BAP and 1 mg/L kinetin.

The phenomenon of albinism is well-known in the in vitro androgenesis of rice [6,8,14,21,22,23,24,25,26,27,35]. In some programs, a high proportion of albinos among the regenerated plantlets reduced the efficiency of DH plantlet production [14,21,22]. The ratio of green and albino plantlets in rice AC is genotype-dependent [24]. Some country-of-origin genotypes (CRHR32, BS6444G) were identified that produced dominantly green plantlets in an improved AC system [11,12], while another country-of-origin genotype (NRVC980385) predominantly produced albinos in experiments performed [14].

Consistent with the findings of other studies [23,24], genotype and medium influenced the number of green and albino plantlets in our experiment. However, this difference (genotype-dependency) was mitigated by modification of the composition of the plant regeneration medium—the ratios of regenerated green and albino plantlets were found to be close to equal for the best treatment in our experiment. More than 1000 green plantlets and approximately 400–500 DH plantlets were acclimatized to glasshouse and field conditions.

### 3.4. Flow Cytometric Analyses, Spontaneous DH Plants and Importance in Plant Breeding

Flow cytometric analyses undertaken confirmed the microspore origin of the haploid calli. The identified diploids were derived from spontaneous DH microspores (predominantly) or possibly from the wall of anthers. The uniformity of the regenerated diploid plants can be checked in the nursery in the DH_1_ and DH_2_ generations. The DH lines from spontaneous DH microspores form uniform populations in micro-plots, while plants from somatic tissue (anther wall) can be segregated in the following generations. The DH lines selected in the DH_1_ generation were found to be uniform in the observational trial.

In our study, the percentage of overall spontaneous chromosome doubling was 42.1%, ranging from 38.1% to 57.9%, depending on genotype. Most studies have obtained similar results (42.24–69%) with respect to spontaneous chromosome doubling [2,5,6,21,24]. However, Cha-um et al. [27] reported lower values (21.5–31.9%).

DH plants are widely used in plant breeding and research [3,36,37] because of their ability to achieve homozygous lines in one generation [38,39]. Rapid-generation advance can accelerate the breeding process enabling up to three to four generations a year, while DH programs can deliver the highest rates of genetic gain [40]. In our experiments, DH plants were regenerated from different generations of sexual crossings. The mean of green plantlet production was 24.48 green plantlets/100 anthers in the best treatment, while the mean spontaneous chromosome doubling was 42.1%. So, a significant number of DH lines could be produced using an in vitro AC protocol, which was influenced by genotype. In the DH_1_ generation, DH lines were selected according to basic agronomic traits and uniformity. The proportion of valuable DH lines was lower among the DH lines derived from the F_1_ generation in comparison with DH lines derived from advanced lines. This is consistent with the findings of Forster and Thomas [41] that the frequency of desired genotypes can be higher in DHs plants derived from later generations of pedigree in-breeding or single seed descent (SSD) programs than from an F_1_ generation. However, the time saved is reduced if the DH lines are derived from a later generation than F_1_. For the DH_2_ generation, selected DH genotypes were tested in advanced yield trials; six lines were found to be promising based on complex agronomic selection. After multilocation field tests, the most promising DH lines will be tested in national trials to register new varieties.

## 4. Materials and Methods

### 4.1. Plant Materials and Growing Conditions

In the experiments, one Hungarian variety (Dáma), one advanced line (Karola), one F_2_ (1009) and 2 F_1_ (Mirko × Karola, Ábel × Dular) combinations of japonica rice, were tested in AC. The donor plants were grown at the Rice Research Station of the Hungarian University of Agriculture and Life Sciences (MATE), Institute of Environmental Sciences (IES), Research Center for Irrigation and Water Management (ÖVKI), located at Szarvas, Hungary. Standard Hungarian rice cultivation technology was used to grow healthy plants in the nursery. Direct seeding was applied on the 28th of April (0 days after sowing; 0 DAS) followed by pre-emergent herbicide spraying (active ingredient: pendimethalin). Permanent flooding was performed from 38 DAS; the irrigation level was set to the development of the plants (5–20 cm). Split nitrogen fertilizer (60–60 kg N/ha) applications (38 and 70 DAS) were used to optimize nutrient management at a total amount of 120 kg N/ha. During the plant growing season, manual weeding was carried out multiple times. The end of the booting stage in the reproductive phase was observed from 75 DAS to 108 DAS, determined by the duration of different varieties and advanced lines.

### 4.2. Collection and Pre-Treatment of Donor Tillers

The donor tillers were collected at the booting stage when the flowers in panicles contained early and mid uninucleated microspores (Figure 1a). The developmental stages of microspores were confirmed with an Olympus CK-2 inverted microscope (Olympus Ltd., Southend-on-Sea, UK). The donor tillers were put into an Erlenmeyer flask containing tap water and were covered with PVC bags to protect the tillers from intensive evaporation under the pre-treatment. The donor tillers were cold pre-treated at 10 °C for three to seven days [25,42,43].

### 4.3. Sterilization and Isolation of Anthers

After pre-treatment, the developmental stages of microspores were rechecked with an Olympus CK-2 inverted microscope (Olympus Ltd., Southend-on-Sea, UK). Panicles suitable for AC were surface sterilized for 20 min in 2% commercial sodium hypochlorite solution with two drops of Tween-80 solution (Duchea Biochemie B.V., Haarlem, The Netherlands). After sterilization, the panicles were rinsed three times with sterile distilled water (Millipore Elix 5). The anthers (100 anthers/Petri dish) were isolated aseptically from the donor panicles and placed in 60 mm diameter plastic Petri dishes (Sarstedt Ltd., Newton, MA, USA) containing different induction media.

### 4.4. In Vitro AC

The N_6_ basal medium [30] was supplemented with growth regulators, 40 g maltose, 500 mg/L L-proline and 500 mg/L L-glutamine, and pH was adjusted to 5.8. The effect of growth regulators (2 mg/L 2,4-D and 0.5 mg/L BAP; 2.5 mg/L NAA, 1 mg/L 2,4-D and 0.5 mg/L kinetin) was tested based on the induction (number of calli) and plant regeneration (green and albino plantlets) by androgenesis (Table 9). The Petri dishes were kept at 28 °C using a thermostat.

### 4.5. Flow Cytometric Analyses of AC-Derived Calli 

The ploidy level of 10 AC-derived calli was determined by flow cytometric analysis (CytoFLEX Flow Cytometer, Beckman Coulter International S.A., Nyon, Switzerland). Samples of friable calli with 3 mm diameter were collected from the plant regeneration medium and callus produced in somatic tissue culture (matured embryo culture) was applied as a diploid control. The samples were chopped with a razor blade with 1 mL Galbraith buffer for 1 min to isolate the nuclei from the samples [44]. The suspensions were purified using 20 µm sieves. DNA content was painted with 40 µL propidium iodide (PI) solution (1 mg/L) for 30 min at 4 °C. After preparation, the DNA content of samples was measured by flow cytometer; the ploidy levels of samples were determined based on histograms.

### 4.6. Plant Regeneration

The AC-derived calli were transferred weekly to 90 mm diameter plastic Petri dishes (Sarstedt Ltd., Newton, MA, USA) containing different plant regeneration media. The collection of AC-derived calli was finished on the 10th week of AC. During the regeneration period, the effects of two basal media (MS and N_6_) and combinations of different growth regulators (1 mg/L NAA, 1 mg/L BAP and 1 mg/L kinetin; 1.5 mg/L BAP, 0.5 mg/L kinetin and 0.5 mg/L NAA) were tested based on the regeneration of green and albino plantlets [30,45]. The MS [45] and N_6_ [30] media were supplemented with the above-mentioned growth regulators and 30 g/L sucrose and pH was adjusted to 5.8 (Table 9).

Well-shooted plantlets were transferred into glass tubes containing half MS medium supplemented with 30 g/L sucrose and pH was adjusted to 5.8 [45]. The green plantlets were cultured in glass tubes until rooting. Well-rooted and shooted green plantlets were transplanted into 720 mL glass jars containing the same half MS medium until transplantation of plantlets to the greenhouse. During the plant regeneration and rooting period, 16 h artificial light (led) and a temperature of 24 °C were maintained in the tissue culture chamber.

### 4.7. Acclimatization of Plantlets

In the first half of March, the well-rooted green plantlets were transferred to the greenhouse and covered with PVC bags to retain adequate humidity. After a five-day acclimatization period, the PVC bags were removed. The green plants were transferred to a paddy field for additional testing. The individual plants were transplanted after permanent flooding was applied. The hill spacing was set to 0.5 m in the nursery. Developmental and morphological characters were observed to identify differences between single plants. The ploidy levels of the regenerated plants were checked by seed set production. The seeds of DH plants were collected after the ripening stage and were integrated into the breeding program.

### 4.8. Field Experiments

The field experiments were carried out at the Rice Research Station of the MATE ÖVKI (Szarvas, Hungary). Genotypes were direct dry-seeded and standard paddy conditions were maintained during the experiments (temporary and permanent flooding until 38–40 DAS and 105–109 DAS, respectively; split nitrogen fertilizer (120 kg/ha); broad-spectrum herbicide (Penoxsulam) at 38–40 DAS; no fungicide). In the DH_1_ generation, the DH lines were tested in a micro-plot experiment (1.5 m rows) with three replications. A total of 58 DH lines were involved in the basic agronomic (observational) trial. Three Hungarian rice varieties (‘Janka’, ‘Dáma’ and ‘M 488’) were used as controls. The DH lines of the 1009 genotype were generated in the tissue culture experiments described here, while the DH lines of Köröstáj and Tünde were produced in an earlier DH plant production program.

The best DH lines were advanced to replicated yield trials (four replications) of DH lines for two years in 2019 and 2020. The yield trials were planted in a randomized complete block design with a 3.6 m^2^ plot size. The evaluation of the lines was performed according to standard breeding criteria (i.e., early vegetative vigor, cold tolerance, blast resistance, earliness, plant height, panicle form, yield and biomass) and completed following the procedures of IRRI [46] and Roumen et al. [47]. Plots were manually harvested; the threshing was performed using a Wintersteiger LD350 laboratory threshing machine (Wintersteiger Co., Ried im Innkreis, Austria). Sartorius BP221S and Sartorius PMA7500 laboratory scales (Sartorius Co., Göttingen, Germany) were used to weigh the samples. Seeds were counted with a Pfeuffer Contador automatic seed counter (Pfeuffer Ltd., Kitzingen, Germany). A Satake THU laboratory husker and a Satake TM05 testing mill (Satake Co., Hiroshima, Japan) were used for the basic milling quality analysis.

### 4.9. Statistical Analyses

The laboratory experiments were carried out in five replications. Data for AC response (i.e., number of calli, albino and green plantlets) were collected and analyzed by two-way ANOVA. The statistical analyses were carried out using Microsoft Excel 2019 statistical software developed by Microsoft (Redmond, WA, USA). Data from the field experiments were statistically analyzed using IBM SPSS ver. 22 software. Three and four plots were used for the DH_1_ and DH_2_ experiments, respectively. Plant phenology and agronomic traits were collected using four replicates. One-way ANOVA with Tukey post hoc test was used to test differences among treatments at the 5% level of probability.

## Figures and Tables

**Table 1:** Statistical analyses of the effect of growth regulators on the number of AC-derived calli.

	df	MS of Calli/100 Anthers
Growth regulators	1	95,922.000 **
Genotype	4	20,521.530 ns
Interaction	4	15,708.150 ns
Error	40	8340.880

** significant at *p* < 0.01. ns, non-significant.

**Table 2:** The effect of different growth regulators on the number of AC-derived calli/100 anthers. The different lower-case letters (a, b) denote significantly different values (*p* < 0.05) between the treatments (LSD_5%_= 52.206), while the different capital letters (A, B, C) denote significantly different values (*p* < 0.05) among the genotypes (LSD_5%_ = 82.559).

Genotype	Karola	Dáma	1009	M × K	Á × D	Mean
N_6_NDK	468.80 a A	264.60 a C	333.60 a BC	376.80 a B	320.00 a BC	352.76
N_6_DB	279.20 b AB	263.20 a AB	203.20 b B	275.20 b AB	305.00 a A	265.16
Mean	374.00	263.90	268.40	326.00	312.50	308.96

**Table 3:** Statistical analyses (two-way ANOVA) of plant regeneration efficiency (number of green- and albino plantlets) using different induction media.

Induction Medium		N_6_NDK		N_6_DB	
	df	MS of Green Plantlets	MS of Albinos	MS of Green Plantlets	MS of Albinos
Regeneration m.	3	1862.187 ***	6801.653 ***	1511.467 ***	2968.320 ***
Genotype	4	8611.440 ***	2334.160 **	5756.560 ***	1178.240 ***
Interaction	12	1046.853 ***	692.987 ns	638.800 ***	190.720 ns
Error	80	189.840	486.160	169.600	164.240

*** significant at *p* < 0.001. ** significant at *p* < 0.01. ns, non-significant.

**Table 4:** Comparison of different plant regeneration media (1, N_6_NBK1; 2, N_6_NBK2; 3, MSNBK1 4; MSNBK2) based on plant regeneration efficiency (number of green and albino plantlets/100 anthers) of AC-culture-derived calli induced on N_6_NDK and N_6_DB induction media. Values followed by corresponding lower-case letters (a, b etc.) are not significantly different (*p* < 0.005) within the same genotype (I, II, III and IV). Values followed by corresponding capital letters (A, B etc.) are not significantly different (*p* < 0.005) within the same treatments.

Number of Green Plantlets/100 Anthers										
Induction medium	I. N_6_NDK					II. N_6_DB				
Regeneration medium	plant regeneration medium					plant regeneration medium				
Genotype	N_6_NBK1	N_6_NBK2	MSNBK1	MSNBK2	Mean of m.	N_6_NBK1	N_6_NBK2	MSNBK1	MSNBK2	Mean of m.
Karola	4.00 ab B	1.60 b B	8.00 ab BC	9.60 a B	5.80	0.00 b B	1.60 b B	9.60 a B	4.80 ab C	4.00
Dáma	0.00 a B	1.60 a B	1.60 a C	0.80 a C	1.00	0.80 a B	0.00 a B	5.60 a B	0.80 a C	1.80
1009	20.80 c A	26.40 c A	95.20 a A	59.20 b A	50.40	21.60 c A	15.20 c A	56.80 b A	72.00 a A	41.40
M × K	6.40 a B	5.60 a B	12.80 a B	10.40 a B	8.80	4.00 b B	6.40 b B	16.00 a B	15.20 a B	10.40
Á × D	0.80 a B	0.00 a B	4.80 a BC	4.00 a BC	2.40	0.00 a B	0.80 a B	1.60 a B	2.40 a C	1.20
Mean of genotype	6.40	7.04	24.48	16.80	13.68	5.28	4.80	17.92	19.04	11.76
Number of albino plantlets/100 anthers										
Induction medium	III. N_6_NDK					IV. N_6_DB				
Regeneration medium	plant regeneration medium					plant regeneration medium				
Genotype	N_6_NBK1	N_6_NBK2	MSNBK1	MSNBK2	Mean of m.	N_6_NBK1	N_6_NBK2	MSNBK1	MSNBK2	Mean of m.
Karola	0.00 b B	0.80 b A	47.20 a A	52.80 a A	25.20	2.40 c B	0.00 c A	10.40 b C	22.40 a B	8.80
Dáma	0.80 b B	0.80 b A	11.20 b B	48.00 a A	15.20	0.80 b B	0.00 b A	22.40 a B	16.80 a B	10.00
1009	2.40 b B	0.80 b A	17.60 a B	10.40 ab C	7.80	4.80 b B	1.60 b A	12.80 a C	14.40 a B	8.40
M × K	32.00 b A	6.40 c A	50.40 a A	48.00 a A	34.20	14.40 c A	7.20 c A	31.20 b A	51.20 a A	26.00
Á × D	4.80 b B	0.80 b A	15.20 ab B	24.80 a B	11.40	0.00 b B	0.00 b A	17.60 a BC	16.00 a B	8.40
Mean of genotype	8.00	1.92	28.32	36.80	18.76	4.48	1.76	18.88	24.16	12.32

**Table 5:** Rate of spontaneous DH plants detected at the nursery field of MATE (Szarvas).

Genotype	Total Green Plants	Fertile DH Plants	Sterile Haploid Plants	Percent of Spontaneous DH Plants (%)
Karola	17	9	8	52.9
Dáma	19	11	8	57.9
1009	444	169	275	38.1
M × K	531	234	297	44.1
Á × D	37	18	19	48.6
Total number of plants	1048	441	607	42.1
Mean of genotype	209.6	88.2	121.4	

**Table 6:** Agronomic traits of DH lines (DH_1_ generation) in micro-scale temperate rice field experiment (Szarvas, Hungary, 2018).

AC-derived DH Lines	Average Biomass	Average Yield	Number of Lines (on Yield Parameters)	
	g/plot	g/plot	Below Control Mean	Above Control Mean
Köröstáj (F_6_)	588.6 (333.3–800.0)	346.9 (208.7–474.0)	11	23
Tünde (F_6_)	654.6 (513.3–773.3)	361.0 (288.3–436.7)	2	6
1009 (F_1_)	638.1 (433.3–933.3)	311.3 (216.0–407.0)	9	7
Control mean	604.8 (483.0–806.7)	326.3 (260.0–459.3)	-	-

**Table 7:** Main phenological and agronomic parameters of anther-derived DH_2_ lines in small-scale field experiment (Szarvas, Hungary, 2019–2020). The different alphabets (a–e) mark significantly different values (*p* < 0.05) among the genotypes.

Anther-Culture-Derived DH_2_ Rice Lines	Plant Height	Biomass	Grain Yield ^1^	Grain Yield	Percent of Control Mean ^2^
	(cm)	(g/parcel)	(g/parcel)	(t/ha)	(%)
1009 42.I. 1	52.6 ± 2.9 ^d^	3761.3 ± 723.4 ^a^	1364.9 ± 124.5 ^a,b^	3.79 ± 0.35 ^a,b^	97.4
1009 22.III. 1	54.0 ± 2.8 ^d^	2981.3 ± 1196.3 ^a,b,c^	1243.0 ± 376.9 ^b^	3.45 ± 1.05 ^b^	88.7
Köröstáj 14.III. 2	53.9 ± 3.8 ^d^	3072.5 ± 329.7 ^a,b,c^	1543.5 ± 132.2 ^a,b^	4.29 ± 0.37 ^a,b^	110.1
Köröstáj 17.II. 1	47.5 ± 3.3 ^e^	2905.0 ± 298.9 ^a,b,c^	1413.9 ± 124.0 ^a,b^	3.93 ± 0.35 ^a,b^	100.8
Tünde 16.II. 2	66.0 ± 3.0 ^a,b^	3010.0 ± 348.3 ^a,b,c^	1479.8 ± 156.3 ^a,b^	4.11 ± 0.43 ^a,b^	105.5
Tünde 25.I. 1	66.4 ± 2.4 ^a,b^	2810.0 ± 363.8 ^b,c^	1459.8 ± 108.0 ^a,b^	4.06 ± 0.30 ^a,b^	104.1
Tünde 16.II. 1	68.5 ± 2.9 ^a^	3086.3 ± 466.2 ^a,b,c^	1564.3 ± 178.9 ^a,b^	4.35 ± 0.50 ^a,b^	111.6
Tünde 17.I. 1	66.0 ± 1.7 ^a,b^	2805.0 ± 261.6 ^b,c^	1343.6 ± 115.6 ^a,b^	3.73 ± 0.32 ^a,b^	95.8
Tünde 14.VIII. 1	70.4 ± 2.3 ^a^	2730.0 ± 372.3 ^b,c^	1391.6 ± 153.5 ^a,b^	3.87 ± 0.43 ^a,b^	99.3
1009 9.II. 1	58.8 ± 2.9 ^c^	3241.3 ± 750.8 ^a,b^	1386.9 ± 268.0 ^a,b^	3.85 ± 0.74 ^a,b^	98.9
M488 control	51.6 ± 1.8 ^d,e^	2327.5 ± 404.9 ^c^	1218.4 ± 246.6 ^b^	3.39 ± 0.69 ^b^	86.9
Janka control	59.4 ± 2.9 ^c^	2621.3 ± 402.9 ^b,c^	1351.5 ± 250.7 ^a,b^	3.75 ± 0.69 ^a,b^	96.4
Dáma control	62.3 ± 2.8 ^b,c^	3126.3 ± 503.8 ^a,b,c^	1636.0 ± 266.8 ^a^	4.55 ± 0.74 ^a^	116.7
Mean of control varieties	57.8	2691.7	1402.0	3.9	100

^1^ Average grain yield of DH_2_ lines (*n* = 8) in a randomized small-scale experiment; ^2^ Percentage of the yield of DH lines compared to the mean of controls.

**Table 8:** Main agronomic and grain quality traits of anther-derived DH_2_ lines in small-scale temperate rice field experiment (Szarvas, Hungary, 2019–2020). The different alphabets (a–h) mark significantly different values (*p* < 0.05) among the genotypes.

Anther-Culture-Derived DH_2_ Rice Lines	Lodging Incidence ^1^	Blast Resistance ^2^	Thousand Kernel Weight	Milling Quality					
				Brown Rice	White Rice (Whole) *		White Rice (Broken) *		Agronomic Selection ^3^
					2019	2020	2019	2020	
	%		(g)	(%)	(%)	(%)			
1009 42.I. 1	0.0	3–4	25.4 ± 1.2 ^h^	77.1 ± 0.9 ^g^	54.1 ± 3.9 ^b,c,d^	59.8 ± 0.7 ^a,b^	11.2 ± 3.2 ^c,d,e^	6.1 ± 1.3 ^f^	+
1009 22.III. 1	0.0	1–2	31.6 ± 0.9 ^d^	79.2 ± 0.7 ^e,f^	59.4 ± 1.0 ^a,b^	57.0 ± 3.0 ^a,b,c^	7.9 ± 0.6 ^e,f^	10.7 ± 3.1 ^c,d,e^	+
Köröstáj 14.III. 2	25.0	6	31.8 ± 1.2 ^d^	78.8 ± 0.9 ^f^	45.6 ± 2.0 ^e,f^	56.8 ± 1.1 ^a,b,c^	19.6 ± 2.0 ^a^	10.9 ± 1.3 ^c,d^	-
Köröstáj 17.II. 1	0.0	1–2	28.1 ± 1.0 ^f,g^	79.2 ± 0.7 ^e,f^	51.0 ± 0.5 ^c,d,e,f^	55.7 ± 1.2 ^b,c^	14.3 ± 0.5 ^a,b,c,d,e^	11.4 ± 0.8 ^c^	+
Tünde 16.II. 2	25.0	3–4	33.4 ± 1.1 ^b,c^	82.4 ± 0.5 ^a,b^	55.3 ± 4.0 ^a,b,c^	62.8 ± 1.0 ^a^	14.3 ± 3.8 ^a,b,c,d,e^	8.5 ± 1.6 ^c,d,e,f^	-
Tünde 25.I. 1	12.5	5–6	33.6 ± 1.3 ^b^	81.8 ± 0.5 ^a,b,c^	60.0 ± 1.9 ^a,b^	62.4 ± 4.0 ^a^	9.3 ± 2.1 ^d,e,f^	6.9 ± 1.9 ^d,e,f^	-
Tünde 16.II. 1	12.5	1–2	33.9 ± 1.1 ^a,b^	82.6 ± 0.9 ^a^	52.1 ± 4.5 ^c,d,e^	61.4 ± 2.0 ^a,b^	17.5 ± 4.3 ^a,b,c^	8.7 ± 1.3 ^c,d,e,f^	++
Tünde 17.I. 1	0.0	1	29.3 ± 0.7 ^e,f^	81.2 ± 0.5 ^c^	54.5 ± 2.2 ^a,b,c^	62.6 ± 1.6 ^a^	14.8 ± 2.1 ^a,b,c,d^	6.8 ± 1.4 ^e,f^	+
Tünde 14.VIII. 1	0.0	5–6	29.6 ± 1.2 ^e^	80.1 ± 0.6 ^d,e^	47.6 ± 2.1 ^d,e,f^	61.0 ± 0.9 ^a,b^	20.8 ± 2.6 ^a^	7.9 ± 0.9 ^c,d,e,f^	-
1009 9.II. 1	0.0	3–4	29.6 ± 0.8 ^e^	77.6 ± 0.6 ^g^	60.9 ± 1.1 ^a^	62.2 ± 5.6 ^a^	4.4 ± 1.2 ^f^	1.8 ± 0.4 ^g^	+
M488 contrl	0.0	5–6	27.5 ± 0.5 ^g^	80.0 ± 0.6 ^e^	53.8 ± 1.0 ^b,c,d^	61.2 ± 1.1 ^a,b^	12.2 ± 1.0 ^b,c,d,e^	7.7 ± 0.6 ^c,d,e,f^	n.a.
Janka control	25.0	6	35.0 ± 0.5 ^a^	81.1 ± 1.2 ^c,d^	45.0 ± 2.6 ^f^	46.1 ± 3.3 ^d^	19.6 ± 2.6 ^a^	21.5 ± 3.0 ^a^	n.a.
Dáma contrl	50.0	1–2	32.1 ± 1.0 ^c,d^	81.5 ± 0.5 ^b,c^	50.7 ± 4.0 ^c,d,e,f^	52.9 ± 1.0 ^c^	17.8 ± 4.1 ^a,b^	15.6 ± 1.3 ^b^	n.a.
Mean of control varieties	-	-	-	80.9	49.8	53.4	16.5	14.9	n.a.

^1^ Evaluation of specific parameters according to IRRI 2014. ^2^ Screening for blast (*Pyricularia oryzae*) resistance according to Roumen et al. 1997. Scale of 1–6 where 1 means resistant, 6 means susceptible reaction. ^3^ Agronomic selection of the DH_2_ lines was performed during the growing season based on complex evaluation of early vegetative vigor, cold tolerance, blast resistance, earliness, plant habit, panicle form and commercial quality parameters (++ high, + moderate, - low performance). *—Seasonal effects were significant only in the case of polished whole and broken white rice. n.a.—not applicable.

**Table 9:** The components of induction, plant regeneration and rooting media in rice AC.

Components of Media (mg/L)	Induction Medium		Plant Regeneration Medium				Rooting Medium
	N_6_DB	N_6_NDK	N_6_NBK1	N_6_NBK2	MSNBK1	MSNBK2	½MS_0_
Basal medium	N_6_	N_6_	N_6_	N_6_	MS	MS	½MS
L-Proline	500	500	-	-	-	-	-
L-Glutamine	500	500	-	-	-	-	-
2,4-D	2	1	-	-	-	-	-
BAP	0.5	-	1	1.5	1	1.5	-
NAA	-	2.5	1	0.5	1	0.5	-
Kinetin	-	0.5	1	0.5	1	0.5	-
Maltose	40,000	40,000	-	-	-	-	-
Sucrose	-	-	30,000	30,000	30,000	30,000	30,000
pH	5.8	5.8	5.8	5.8	5.8	5.8	5.8
Gelrite	2800	2800	2800	2800	2800	2800	2800

## Data Availability

All data used in this manuscript are presented in the manuscript.

## Abbreviations

2,4-D	2,4-Dichlorophenoxyacetic acid
AC	Anther culture
BAP	6-Benzylaminopurin
DH	Doubled haploid
IAA	Indol-3-acetic acid
NAA	1-Naphtylacetic acid
MS	Murashige and Skoog
DAS	Days after sowing
